# Gratitude as a Protective Factor for Cyberbullying Victims: Conditional Effects on School and Life Satisfaction

**DOI:** 10.3390/ijerph18052666

**Published:** 2021-03-09

**Authors:** Xavier Oriol, Jorge Varela, Rafael Miranda

**Affiliations:** 1 Escuela de Educación, Facultad de Educación y Ciencias Sociales, Universidad Andres Bello, Santiago 7550000, Chile; xavier.oriol@unab.cl; 2 Facultad de Psicología, Universidad del Desarrollo, Santiago 7610658, Chile; jovarela@udd.cl; 3 Departamento de Psicología, Facultad de Humanidades, Universidad Continental, Lima 15046, Peru

**Keywords:** cyberbullying, overlap between forms of bullying, school satisfaction, life satisfaction, dispositional gratitude

## Abstract

Recently, studies linking the emotion of dispositional gratitude to cyberbullying have attracted attention. However, this is still a seminal research area that requires further scientific studies. Through longitudinal data, this study aims to analyze the mitigating effect of gratitude on cybervictimization and two indicators of adolescent subjective well-being, namely school and life satisfaction. To this end, 221 adolescents attending private schools in Peru (age: mean (M) = 12.09; standard deviation (SD) = 0.89) were selected to respond to a self-administered questionnaire in two waves that were six months apart. Descriptive data show that 27% of cybervictims also suffer other types of traditional bullying. The overlaps between forms of bullying contribute to higher probabilities of experiencing low school and life satisfaction compared to non-victims after six months. The results of the moderation analysis show that experience high gratitude help students to maintain stable levels of life satisfaction regardless of the prevalence of cyberbullying after six months The results are discussed in terms of the relevance of fostering gratitude from early ages.

## 1. Introduction

Bullying is considered to be a public health problem that affects children and adolescents in countries around the world [1]. The first studies on bullying focused on face-to-face violence [2], but over recent years, interest in the phenomenon of cyberbullying, which implies aggression through electronic devices, has increased (e.g., [3]). The prevalence of this form of bullying is usually lower than that of traditional bullying according to various meta-analyses [4,5], although cyberbullying prevalence can increase due to the widespread use of social networks.

In Latin America, a higher prevalence of traditional bullying across countries is usually observed. However, a recent study conducted by Herrera-López, Romera and Ortega-Ruiz [6] shows that the prevalence of cyberbullying is close to that of other European countries and the USA. Among Latin American countries, Peru presents one of the highest prevalence rates of traditional bullying (e.g., [6,7]). This is due to the structural violence present in different areas and contexts of the Peruvian society, especially affecting women, children and adolescents [8,9]. In some regions of the country, violence from adults to children and adolescents is still common, and this practice is considered necessary for correcting disruptive behavior [8,10]. The high prevalence of structural violence directly influences school violence between peers, which makes it a key aspect for the prevention of violence in Peru [9].

Regarding the prevalence of cyberbullying, a recent study with 47,114 Peruvian adolescents indicated only a prevalence of 8.1% of this type of violence among peers [11]. Nevertheless, the low levels observed could be closely related to the low rate of access to the Internet in many regions of Peru [12]. In this sense, other data on cyberbullying gathered from 5771 adolescents attending 71 schools in Lima show a much higher prevalence of up to 31.3% [9].

### 1.1. Cyberbullying and Its Overlap with Other Forms of Traditional Bullying

Children and adolescents today have almost universal access to different types of technologies, such as mobile phones, iPads and the Internet and to various social media platforms. This access allows them to participate in new and more complex communities that extend beyond the schoolyard. However, this new digital context has led to the presence of aggressive episodes between students and associated negative consequences. Accordingly, researchers have attempted to examine these negative aggressive events in these digital settings [13]. Researchers have labeled this aggressive behavior as cyberbullying due to the overlap between bullying at the school and bullying suffered online [14]. Cyberbullying can be characterized as a type of bullying behavior that makes use of technologies, mostly because there is an important interrelation between the students who are involved in bullying and cyberbullying [15]. Cyberbullying has been defined by Smith et al. [16] as “an aggressive, intentional act carried out by a group or individual, using electronic forms of contact, repeatedly and over time against a victim who cannot easily defend him or herself” (p. 376). Nevertheless, recent studies have questioned the need to consider cyberbullying as a repetitive behavior for the calculation of its prevalence (for review, [17]). Cyberbullying is also different from traditional bullying because it not only does it take place via electronical devices but it is also a form of interpersonal violence that occurs outside of school [2,18].

The prevalence of this phenomenon, as stated above, seems to be lower than the prevalence observed for traditional bullying at the global level [4,19]. Despite this, there are still important discussions regarding how to calculate this prevalence due to the differences between the instruments used to assess cyberbullying [17]. Another calculation-related problem in Latin American countries such as Mexico, Peru and Chile is the lack of awareness of this form of violence among peers as a problem and issue that needs to be addressed [20].

Another additional aspect to consider in the study of cyberbullying is that many bullies and cybervictims have also suffered some kind of traditional bullying [2]. Victims of cyberbullying itself show more symptoms of internalization and externalization than victims of traditional bullying [2]. However, when cybervictimization occurs simultaneously with other forms of face-to-face aggression, polyvictimization by peers is experienced, which increases the risk of anxiety, depression or loneliness, among other psychological problems [21]. Furthermore, having suffered different forms of victimization at once is a stronger predictor of mental health outcomes than having experienced only one form of victimization repeatedly [22]. Recently, it has been observed that suffering an overlap between traditional and bullying leads to a decrease in the subjective well-being of adolescents [11].

### 1.2. Effects of Cyberbullying Victimization on School and Life Satisfaction

As observed over the last 10 years, an increasing number of studies have focused on the consequences of bullying through electronic devices on the mental health of adolescents (for a review, see [23,24]). Nevertheless, fewer studies have analyzed the relationship between this type of peer victimization and the subjective well-being indicator [11], although this construct is considered fundamental to assess adolescent adjustment [25]. In this sense, subjective well-being is defined as the subjective global appraisal that people make of their own lives and is formed by a cognitive component (referred to as the cognitive appraisal of life satisfaction) and by an affective component (the experience of negative affects and the absence of negative affects) [26]. In addition to these components, interest in school satisfaction has also increased recently, as this is a relevant indicator of subjective well-being in adolescents due to the time they spend at school [27,28]. In other studies, the need to consider school satisfaction as a subjective well-being indicator has been discussed, since a strong relationship is observed between life satisfaction and other single-item and multi-item scales that assess, for instance, life satisfaction [7,27,28], and because school is the second most important socialization context after family at this age [7,29]. School satisfaction was initially defined as the appraisal of the generally positive aspects [29,30]. In this sense, the initial Multidimensional Students Life Satisfaction scale proposed by Huebner [30], which has been one of the most used tools in this context, comprises different items about aspects such as satisfaction with learning at school and interest in school and in its activities. Subsequently, other studies have also incorporated other items about satisfaction with interpersonal relationships with teachers or friends from school [7,27].

The existing research into the effects of cyberbullying on subjective well-being indicators such as life and school satisfaction shows that adolescents that are victims of this form of bullying present lower satisfaction than non-victims [31,32,33], and the fact of suffering cyberbullying also has a negative relationship with school satisfaction [34,35,36,37]. Recently, it has been observed that an overlap between traditional bullying forms and cyberbullying increases the probabilities of experiencing a decrease in life satisfaction during adolescence [11]. In this sense, more studies are required to delve into the relationship between these variables, considering that an overlap between bullying forms is common during adolescence [2,11]. In addition to this, there is a lack of more longitudinal studies that analyze the prospective relationships among these variables. An increase in the literature in this field could allow for a better understanding of the factors that can prevent and moderate the relationship between cybervictimization in order to relieve its effects on adolescent well-being [9].

In summary, despite the increase in studies linking cyberbullying and subjective well-being indicators over the past decade, more studies are still required to observe the longitudinal relationships between these variables in adolescent samples. In addition, further studies delving into this relationship while considering the effect of suffered cyberbullying on subjective well-being indicators when the victims also experience other kinds of traditional bullying simultaneously are also required. 

### 1.3. Dispositional Gratitude as a Protective Factor against Cybervictimization

Gratitude is an emotion elicited when people are helped by others and is usually considered to be a trait [38]. Thus, to measure this emotion, some of the most used scales ask people about the frequency at which they experience gratitude in their everyday life [39,40]. From an evolutional point of view, gratitude is considered to be one of the emotions that contributes most to the creation of stable bonds to kin, non-kin and social collectives, and thus it belongs to the family of moral emotions [41]. More recently, in addition to compassion and awe, gratitude has been placed in the category of self-transcendent emotions, which were given this denomination since they promote prosociality towards other people [42,43].

Gratitude is an emotion that promotes prosocial behavior towards others, as well as a constant positive affect [44,45]. According to the broaden-and-build theory of positive emotions, the accumulation of positive affective experiences can be an important factor to promote subjective well-being. Specifically, the experience of a positive affect would also act as a mediator between gratitude and life satisfaction [46]. Other studies have also indicated a relationship between gratitude and the cognitive component of well-being related to life satisfaction. For example, a study conducted by Datu and Mateo [47] with 409 Philippine adolescents showed that gratitude was positively associated with life satisfaction, as well as mediated partly by meaning in life. Another recent study conducted by You, Lee, Lee and Kim [46] with 877 Korean teenagers indicated that grateful students experienced higher satisfaction. On the contrary, gratitude has been negatively associated with mental health problems such as anxiety and depression during adolescence [47].

Gratitude has also been shown to have a strong relationship with another important indicator for subjective well-being in adolescence: school satisfaction [48,49]. In a study conducted by Froh, Yukewicz and Kashdan [50], gratitude was observed to present a robust relationship with school satisfaction. In this sense, other studies have also indicated that interventions promoting gratitude contribute to an increase in school satisfaction [51].

Additionally, gratitude has been demonstrated to be a crucial protective factor for both preventing bullying [52] and cyberbullying [53]. Recently, its mitigating effects on mental health problems have also been studied, for example, in cases of adolescent suicide [54]. Experiencing constant gratitude helps people to deal better with negative experiences (e.g., [55]), and therefore this construct can be a key factor for the mitigation of the effects of bullying. In this sense, given the strong relationship between gratitude with life and school satisfaction, this trait emotion may have a mitigating effect on its relationship with cyberbullying victimization.

To summarize gratitude is a dispositional emotion that has a robust relationship with adolescent life and school satisfaction [37,46]. Additionally, the constant experience of gratitude may help adolescents to deal better with negative experiences [54,55]. However, more studies on the moderating role of gratitude on adolescent well-being indicators for victims of bullying and cyberbullying are necessary (e.g., [53]). In this sense, the hypothesis that gratitude moderates the relationship between cyberbullying and life and school satisfaction is proposed.

### 1.4. Present Study

Most studies about cyberbullying have focused on its consequences for mental health [11]. However, in recent years, there has been a growing interest in relating this form of bullying with well-being indicators including life and school satisfaction [11,32]. In this line, different studies have demonstrated that cyberbullying victimization is negatively related to different adolescent well-being indicators such as school and life satisfaction (e.g., [31,32,33,34]). Nevertheless, few works have considered this relationship when cyberbullying victims also suffer other forms of traditional bullying [11].

Furthermore, there is a need for more longitudinal studies into the prospective relationships between these variables. In this context, a longitudinal methodology was used in this work, which was applied in two waves six months apart from each other in orther to 1) Assess the effect of suffering cybervictimization and other forms of traditional bullying simultaneously on school and life satisfaction. Thus, we developed hypothesis

**Hypothesis** **1** **(H1):** *Cybervictims who have also suffered other forms of victimization are expected to have increased possibilities of having lower levels of school and life satisfaction than non-victims.*

In another vein, a key aspect in these studies is to understand what protective factors can mitigate the effect of this form of victimization on well-being indicators [9,11]. In this sense, some recent studies have demonstrated that dispositional gratitude can be a crucial factor in the prevention of cyberaggression [53]. However, little is known about the protective effect that this can have on bullying and cyberbullying victims. Since the experience of gratitude reduces the risk of suicidal ideation in adolescents and helps them to cope with negative experiences in a better way [54,55], it may exert a moderating effect on the prospective relationship between cybervictimization and life and school satisfaction. In this sense,

**Hypothesis** **2** **(H2):** *Gratitude will have a moderating effect on the relationship between cyberbullying victimization and school satisfaction.*

**Hypothesis** **3** **(H3):** *In the same line, propose that gratitude will have a moderating effect on life satisfaction.*

## 2. Materials and Methods

### 2.1. Participants

The sample of this study was taken from data collected for a project on the assessment of social–emotional skills, called “KUSI”, which was implemented in private educational institutions in Lima (Peru). Data were collected in two waves, which were six months apart from each other.

The implementation of KUSI was comprised of all the students attending the sixth grade of primary school and first and second year of secondary school. Therefore, it was not necessary to select a sample for the study.

In total, 360 students participated in the data collection. Of this total, 292 students were part of the baseline and 270 were part of the final evaluation of the project. Comparing both groups, 235 students responded to both the baseline questionnaire and the final evaluation of the project. Of these 235 participants, 14 did not answer more than 10% of the questions related to the evaluated scales. In the end, the sample used for the analysis was composed of 221 participants.

At the global level, the mean age of participants was 12.09 (SD = 0.89) for the baseline and 12.54 (SD = 0.93) for the final assessment; likewise, the distribution by grade was 37.8% for the sixth grade of primary school, 36% for the first grade of secondary school and 26.2% for the second grade of secondary school.

### 2.2. Procedure

Participation was voluntary and confirmed with an informed consent form that was signed by guardians and given with the assent of the students. The study was approved by the Ethics Committee of Continental University (ethical approval code: 004-2019-CE-FH-UC; approval date: April 2019).

A letter explaining the objective of the program and the survey was sent to the students’ guardians. If they agreed with the participation of their child, they had to sign and turn the informed consent form. In the case of student assent, students were asked to write down in their names on the assent form if they wanted to take part in the project.

The application of the instrument was conducted through a web form on the SurveyMonkey platform. The evaluation was conducted simultaneously in all sections that were part of the project. A personalized link was sent to students to identify the origin of the questionnaire answers and pair them with the corresponding information from the database.

Students that formed part of the evaluation completed the questionnaires using their personal laptops. Those students that did not want to take part in the study were taken to a separate room during the application of the survey. The average test time was about 35 min.

The program was implemented between May (T1) and November (T2) 2019 and the evaluations were conducted with the same cohort in both months.

### 2.3. Instrument

A battery of scales from the Single School Well-being Questionnaire developed by the Ministry of Education of Peru [56] was employed for data collection. this questionnaire comprises different scales in three dimensions: (1) socioemotional skills, (2) school climate and (3) a school violence scale. Cronbach’s alpha (α) and McDonald’s omega (Ω) were used for both the initial evaluation (T1) and the final evaluation (T2). In the reliability analysis, alphas are considered acceptable at (0.66) and good at (0.80) according to the internal consistency criteria established by Kline [57] Likewise, confirmatory factor analysis (CFA) indexes were presented for both scales.

- Life Satisfaction: The Satisfaction with Life Scale for Children developed by Gaderman, Guhn and Zumbo [58] was used to measure this construct. This is composed of five items (e.g., “In many senses, my life is similar to the life I’d like to have”). Answers were assessed with a five-point Likert scale in which 0 represented “never”, 1 represented “seldom”, 2 represented “sometimes”, 3 represented “usually” and 4 represented “always”. To assess instrument reliability, the indicators were calculated and yielded the following values: Ω_T1_ = 0.87 and Ω_T2_ = 0.87, as well as α_T1_ = 0.87 y α_T2_ = 0.86. In the case of adjustment indicators for the confirmatory factor analysis (CFA), the values were a comparative fit index (CFI) of 0.99, Tucker–Lewis index (TLI) of 0.99 and root mean square error of approximation (RMSEA) of 0.06 for T1, and a CFI of 0.99, TLI of 0.98 and RMSEA of 0.07 for T2.
- School Satisfaction: The scale developed by Long, Huebner, Wedell and Hills [59] was used, which is based on the instrument for school satisfaction in adolescents and has five items (e.g., “I learn quite a lot at school”). Answers were assessed with a five-point Likert scale in which 0 represented “never”, 1 represented “seldom”, 2 represented “sometimes”, 3 represented “usually” and 4 represented “always”. Regarding the adjustment indicators of CFA, a CFI of 0.96, TLI of 0.91 and RMSEA of 0.15 were found for T1, and a CFI of 1.00, TLI of 1.00 and RMSEA of 0.02 were found for T2. The reliability indicator values were as follows: Ω = 0.86 and α = 0.86 for T1 and Ω = 0.89 and α = 0.89 for T2.
- Cyberbullying Victimization: This scale was composed of four items (e.g., “I have been excluded or ignored from a social network or chat by a group of students”). It originated from a scale denominated as the National Survey of School Violence develop by the Minister of Education in Peru [60]. Each item addressed the prevalence of virtual violence that a student had suffered over the past month. This scale was assessed though a four-point Likert scale, where 0 represented “never”, 1 represented “once”, 2 represented “two to three times” and 3 represented “four or more times”. Regarding the adjustment indicators of CFA, a CFI of 0.91, TLI of 0.80 and RMSEA of 0.09 was found for T1, with the best reliability indicators at T1 being an Ω of 0.85 and α of 0.60, whereas these values were a CFI of 0.97, TLI of 0.92 and RMSEA of 0.11 for T2. Reliability values were an Ω of 0.82 and α of 0.81.
- Bullying Victimization: This scale was composed of five items that measured the prevalence of direct aggressions toward students (e.g., “A student or a group of students beat me, kicked me or pushed me”). This scale was selected from the National Survey of School Violence by MINEDU [60] and assessed the prevalence of aggression during the last month. This scale was based on a four-point Likert scale, where 0 represented “never”, 1 represented “once”, 2 represented “two to three times” and 3 represented “four or more times”. Regarding the adjustment indexes of CFA, T1 showed a CFI of 0.99, TLI of 0.99 and RMSEA of 0.12, while T2 showed a CFI of 0.96, TLI of 0.90 and RMSEA of 0.09. In terms of reliability indicators, they were an Ω of 0.65 and α of 0.60 for T1, and an Ω of 0.61 and α of 0.63 for T2.
- Dispositional Gratitude: This scale was developed by Froh, Emmons, Card, Bono and Wilson [61] and formed by four items from the gratitude questionnaire for adolescents, using a five-point-Likert scale where 0 represented “Totally disagree” and 4 represented “Totally agree”. It contained items such as “I like to return favors” and “I appreciate other people helping me”, among others. Regarding reliability indicators, an Ω of 0.84 and α of 0.85 were found for T1, and an Ω of 0.79 and α of 0.78 were found for T2. Finally, the adjustment indexes of CFA were a CFI of 1.0, TLI of 1.0 and RMSEA of 0.01 for T1, and a CFI of 0.99, TLI of 0.97 and RMSEA of 0.07 for T2.

## 3. Analysis Plan

Before performing the calculations related to the objectives of this work, a confirmatory factor analysis (CFA) was conducted for each scale using the software AMOS v.22. To calculate factors, the robust maximum likelihood estimator (MLR) was employed. Regarding the adjustment indexes, those suggested by Marsh et al. (2004) and by Hu and Bentler (1998) were employed. These authors recommended a Tucker–Lewis index (TLI) and a comparative fit index (CFI) equal to or higher than 0.90, and a root mean square error of approximation (RMSEA) lower than 0.08. To calculate reliability, Cronbach’s alpha (α) and McDonald’s omega (Ω) were used for both the initial evaluation (T1) and the final evaluation (T2). For this analysis, alphas are considered acceptable at (0.66) and good at (0.80) according to the internal consistency criteria established by Kline [62].

Subsequently, descriptive analyses were performed on the sociodemographic variables, including t-Student, Chi square and ANOVA, to compare variables by gender. Likewise, Pearson correlation analyses were carried out between variables.

For Hypothesis 1, a cluster analysis was employed to classify life and school satisfaction. This ordinal classification—low, medium and high—was conducted using Kmeans through SPSS v22. From cluster segmentation, a logistic regression was conducted to measure the reported effect of cybervictimization and also some kind of traditional bullying compared to not suffering any type of aggression on life and school satisfaction. To calculate this effect, logistic regression was used to calculate the odds ratios corresponding to an interval level (confidence interval (CI)) of 95%. In turn, to calculate the odds ratio, the life satisfaction was segmented through cluster analysis. The first cluster (*n* = 29, cluster center = 1.39) corresponded to low levels of life satisfaction, the second one (*n* = 99, cluster center = 2.60) corresponded to the medium level of life satisfaction and the third cluster to students to the higher life satisfaction (*n* = 94, cluster center = 3.62). In the case of school satisfaction, the same procedure was conducted using a K-means cluster; for this variable, the low, medium and high levels of school satisfaction had cluster centers at 1.27 (*n* = 11), = 2.65 (*n* = 74) and 3.72 (*n* = 137).

For Hypotheses 2 and 3, moderation analyses were performed using the PROCESS macro of SPSS v23 [62]. PROCESS uses ordinary least squares and a bias-corrected bootstrap method (with 5000 bootstrapped samples) to estimate conditional effects. This study employed model 1 from Preacher, Rucker and Hayes [63].

For the calculation of moderation, the means of the variables analyzed were previously centered. Regarding the model interactions, probe interaction was set at *p* < 0.05; likewise, conditional values were SD − 1, mean and SD + 1 of the levels of moderation employing the mean from the Johnson–Neyman technique.

## 4. Results

Table 1 presents comparisons of the main sociodemographic variables by gener. In this table, males can be seen to have a higher mean age than females; likewise, there is a higher percentage of males in all grades assessed.

Finally, regarding the mean of best friends, males presented higher scores than females for both the baseline and evaluation.

Regarding Hypothesis 1, Table 2 presents the odds ratios (ORs) of those who suffered cyberbullying and also other types of traditional bullying at T1 over life and school satisfaction at T2. Concretely, 27% of cyberbullying victims in the sample suffered also at least one type of traditional bullying.

The results show that cyberbullying victims who simultaneously suffered other types of traditional bullying were more prone to be part of the group that experienced lower life satisfaction (OR = 1.35, CI (0.20–3.09). Additionally, in the case of school satisfaction, victims were observed to have odds of 1.22 of experiencing low levels of school satisfaction compared to those who did not suffer aggression.

Table 3 presents descriptive indicators associated with each of the variables. At the general level, indicators related to life satisfaction presented higher levels at T1 compared to T2; the same tendency was observed for gratitude. In the case of cyberbullying, the indicator remained the same for both T1 and T2.

Regarding the correlations, the results were mixed. One part of the relationships was found to be significant at a weak and moderate level [64] and another part of the variables did not present significant correlations.

Regarding Hypothesis 2, Table 4 shows the results of the interaction between dispositional gratitude (T1) and cyberbullying (T1). Model 1 incorporates dispositional gratitude (T1) and cyberbullying (T1) separately. Values are B = 0.02, *p* > 0.05 for dispositional gratitude and B = −0.76, *p* < 0.05 for cyberbullying. In Model 2, the moderator variable of dispositional gratitude and cyberbullying is included, which is significant (B = 0.77, *p* < 0.05).

**Figure 1:**
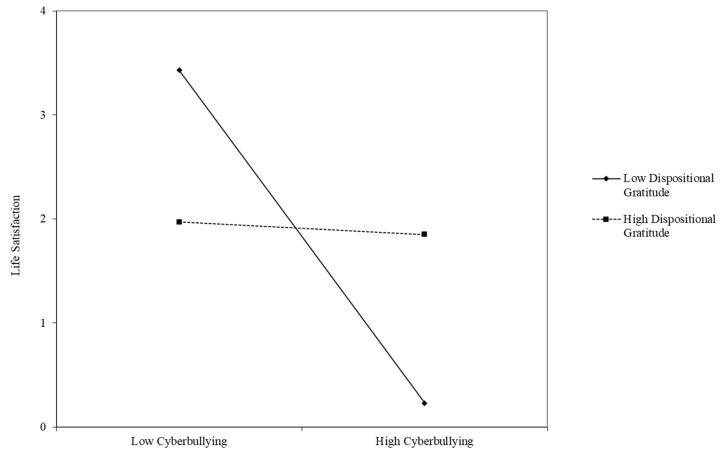
Regression and interaction between life satisfaction and dispositional gratitude considering low and high levels of cyberbullying.

Figure 1 presents the moderation of gratitude and cyberbullying over life satisfaction. As observed in the graph, the slope of the regression is much steeper in those students with lower gratitude levels, meaning that these students would be more prone to a decrease in their life satisfaction when experiencing high levels of cyberbullying. Additionally, in the case of the group with high gratitude levels, life satisfaction was observed to be at stable levels regardless of the prevalence of cyberbullying.

Regarding Hypothesis 3, Table 5 presents the results of the moderating effect of cyberbullying (T1) and dispositional gratitude (T1) on school satisfaction (T2) as a dependent variable. In Model 1, dispositional gratitude and cyberbullying are evaluated separately. The values were B = 0.16, *p* < 0.05 for dispositional gratitude, and B = −0.41, *p* < 0.05 for cyberbullying. In Model 2, dispositional gratitude can be seen to have a significant effect on school satisfaction, with values of B = 0.17, *p* < 0.05; however, the interaction between cyberbullying and dispositional gratitude was not significant, at B = 0.29, *p* > 0.05.

## 5. Discussion

As expressed in the international literature, cybervictims often also suffer other types of face-to-face bullying simultaneously [2]. In relation to that, the descriptive results of this study show that 27% of cyberbullying victims reported having suffered other types of traditional victimization simultaneously. These data are relevant because cyberbullying victims who suffer other types of victimization present higher levels of depression, suicidal ideation, substance use disorders and psychological distress [22,65], as well as lower subjective well-being [11].

Therefore, the first objective of the study was to observe the longitudinal effect of suffering cybervictimization and also other kinds of traditional bullying on two subjective well-being indicators (life satisfaction and school satisfaction) compared to non-victim adolescents. Therefore, the hypothesis proposed is that victims were expected to experience lower levels of school and life satisfaction.

The results show that adolescents who suffer cybervictimization and other types of traditional bullying simultaneously have a greater probability of exhibiting lower levels of life and school satisfaction than non-victims. As commented above, these data are in agreement with previous data that show the negative mental health repercussions of suffering cyberbullying and other types of face-to-face aggressions [22,66]. It should also be considered that adolescence is a period in which there is a systematic decrease in subjective well-being, especially from 11 years of age, which makes teenagers much more susceptible to different mental health problems [67,68]. Thus, schools should promote the prevention of peer violence in order to stop further decreases in well-being and a subsequent increase in mental health problems. Considering the relevance of the school domain to subjective well-being during adolescence [28,68], suffering peer victimization and a reduction in school satisfaction can clearly become significant risk factors at these ages.

In this sense, the second hypothesis in this work was developed to observe the moderating effect of gratitude on the relationship between cyberbullying victimization and life satisfaction, specifically the moderating effect of gratitude on this relationship. The results obtained show first a strong negative relationship between cyberbullying and life satisfaction, but no significant relationship was observed between gratitude and life satisfaction. Despite this fact, gratitude does have a mitigating effect. The group of cyberbullying victims with a low prevalence of bullying and higher gratitude exhibited lower life satisfaction than victims with a low prevalence of bullying and low gratitude. This a priori result is unexpected because the experience of gratitude is usually strongly related to life satisfaction in adolescence [48,49]. Nevertheless, the results fir the conditional effect also indicate that when people suffer a high prevalence of cyberbullying, their life satisfaction only be slightly decreased due to gratitude, as opposed to victims who experience a high prevalence of cyberbullying and low gratitude, who suffer a sharp decrease in life satisfaction. Finally, it must be noted that, as the R2 of the model is relatively low and the size of the sample is not excessively large, the effects need to be interpreted with caution.

In this sense, based on the results, gratitude might act as a protective factor for cyberbullying and prevent an excessive decrease in life satisfaction. According to previous studies, people with high gratitude are characterized as having a broad social support network as this emotion promotes the maintenance of interpersonal relationships [39,69], as well as the accumulation of a constant positive affect [42]. Therefore, the experience of gratitude could encourage people to seek social and emotional support from others, which would mitigate the effect of cyberbullying on life satisfaction, as our results indicate.

In the same line, the third hypothesis of the study sought to determine whether gratitude exhibited a moderating effect on the relationship between cyberbullying and school satisfaction. The results show a strong negative relationship between cyberbullying and school satisfaction; however, the interaction between cyberbullying and gratitude in this case is not significant, and consequently gratitude cannot be said to exhibit a longitudinal moderating effect. One of the possible explanations for our results is that, since the indicator of school satisfaction measures the perception of adolescents about their school experience, although teenagers may perceive more support or even have more personal resources, their perception of school satisfaction does not necessarily improve if cyberbullying is still present in the peer group and the school does not take measures to reduce or prevent it. In this sense, all members of the school community play a crucial role in providing enough support to cyberbullying victims, so they can recover positive perception of the school experience.

Furthermore, the results of this model also show that the relationship between cyberbullying and school satisfaction is less strong than the relationship between cyberbullying and life satisfaction observed in the first moderating model. In this sense, it must be noted that in contrast with traditional bullying, cybervictimization occurs through social networks and therefore is not closely related to the time that an adolescent spends at school, as is the case with traditional bullying [70,71]. This may explain the fact that this type of victimization seems to affect the global indicator of life satisfaction more than that of school satisfaction.

## 6. Conclusions

The descriptive data from the study shows the repercussions of the overlap between traditional forms of victimization and cyberbullying on two fundamental indicators of adolescent subjective well-being, namely school and life satisfaction. Besides this, gratitude appears as a protective factor that mitigates the effect of cyberbullying on life satisfaction, but no moderating effect is observed in the case of school satisfaction.

As shown by the recent review conducted by Zych, Baldry and Farrington [72] on the effectiveness of cyberbullying prevention programs, this type of victimization requires the development of skills to navigate the network safely and train teachers and parents in the external monitoring of the use of social networks. This represents a major challenge in countries such as Peru, where there is still a lack of public awareness about the existence of this type of victimization and its consequences. In this sense, it is first necessary for the educational community becomes aware that cyberbullying is a form of victimization through electronic devices and its main characteristics and consequences on well-being and mental health are known. Second, as indicated by the results of this study, it is advisable to support programs and actions that allow the cultivation of moral emotions such as gratitude from early ages, as they are a key element in the fight against these forms of victimization. Since this is a form of aggression that occurs online, bullies often depersonalize victims, which promotes moral disengagement.

In this sense, dispositional gratitude implies the development of a constant assistance attitude that enables stability in the development of interpersonal relationships and constantly increases positive affects through prosocial behavior [39]. Thus, it may contribute to the prevention of cyberbullying behavior and be a protective factor for victims due to the personal resources provided by the constant experience of this emotion.

## 7. Limitations

It must be noted that this study included a small sample of adolescents, so it is important to conduct more research on the longitudinal relationships between these types of variables. Moreover, the sample included adolescents from private schools, and therefore these results should be replicated in public school contexts in Peru, where there is a higher prevalence of traditional forms of bullying and cyberbullying. The private school sample was from the metropolitan region; consequently, more studies are required in other Peruvian regions where there is sometimes no awareness about cyberbullying as a problem to be studied. It should be also noted that the R2 in the moderation model of gratitude over life satisfaction was quite low, which implies that the results need to be interpreted cautiously. Therefore, more longitudinal studies with larger samples are required to explore the type of relationship between the variables.

Finally, it is worth noting that this study deals with the two most cognitive indicators of subjective well-being: life satisfaction and school satisfaction. For future research, it would be interesting to use both cognitive and affective scales.

## Figures and Tables

**Table 1:** Demographic variables of students by gender.

Variables	Phase	Boys	Girls	t or χ^2^
1. Age	T1	12.2 (0.93)	11.9 (0.77)	2.95 **
	T2	12.7 (0.96)	12.3 (0.83)	2.76 **
2. Grade				
Sixth of primary school		34.3%	39%	7.40 *
First of secondary school		32.1%	15.9%	
Second of secondary school		33.6%	45.1%	
3. Number of siblings	T1	1.5 (0.86)	1.3 (0.5)	2.07
	T2	1.6 (0.87)	1.3 (0.52)	2.78 *
4. Health				
Bad	T1	0%	0%	2.57
Regular		8.7%	6%	
Good		60.9%	71.4%	
Excellent		30.4%	22.6%	
Bad	T2	0.7%	0%	4.49
Regular		7.1%	8.5%	
Good		57.9%	69.5%	
Excellent		34.3%	22%	
5. Number of best friends	T1	7.78 (10.51)	4.83 (3.83)	2.43 **
	T2	7.32 (8.39)	4.76 (3.39)	3.15 **

Note: ** *p* < 0.0, * *p* < 0.05.

**Table 2:** Logistic regression comparing cyberbullying victims who also suffered other kinds of traditional bullying with non-victims over life and school satisfaction. OR: odds ratio; CI: confidence interval; SE: standard error.

	Life Satisfaction (T2) ^a^		*p*-Value	School Satisfaction (T2) ^b^		*p*-Value
	OR (95% CI)	SE		OR (95% CI)	SE	
Cybervictims ^+^ (T1)	1.35 (0.20–3.09)	0.33	0.001	1.22 (0.50–2.09)	0.22	0.03
No aggression						

**^+^** This variable is based on those students who suffered cyberbullying and an additional some type of traditional bullying. **^a^** Dependent variable = life satisfaction, ^b^ Dependent variable = school satisfaction.

**Table 3:** Descriptive analysis and correlations among all the variables for T1 and T2.

Variables	Mean (DE)	Range	1	2	3	4	5	6	7	8
1. Life satisfaction (T1)	3.02 (0.75)	0–4								
2. Life satisfaction (T2)	2.87 (0.82)	0–4	0.50 **							
3. School satisfaction (T1)	3.26 (0.69)	0–4	0.37 **	0.18 **						
4. School satisfaction (T2)	3.24 (0.74)	0–4	0.15 *	0.32 **	0.45 **					
5. Cyberbullying (T1)	0.14 (0.26)	0–3	−0.16 *	−0.24 **	−0.15 *	−0.14 *				
6. Cyberbullying (T2)	0.14 (0.30)	0–3	−0.04	−0.13 *	−0.04	−0.09	0.26 **			
7. Dispositional Gratitude (T1)	3.41 (0.61)	0–4	0.29 **	0.004	0.36 **	0.13 *	0.05	−0.10		
8. Dispositional Gratitude (T2)	3.35 (0.55)	0–4	0.06	0.23 **	0.08	0.29 **	0.07	−0.06	0.32 **	-

Note: Cronbach’s alpha coefficients are presented in parentheses. ** *p* < 0.0, * *p* < 0.05.

**Table 4:** Linear regression models for life satisfaction (Model 1) and Hayes linear regression considering dispositional gratitude as a moderator variable (Model 2).

Dependent Variable = Life Satisfaction (T2)	Model 1		Model 2	
	B	t	B	t
Cyberbullying (T1)	−0.76 **	−3.58	−0.83 **	−3.9
Dispositional Gratitude (T1)	0.02	0.24	0.04	0.4
Cyberbullying (T1) × Dispositional Gratitude (T1)			0.77 *	2.03
R2	0.06		0.07	
F (df1,df2)	6.42 (2.219)		5.71 (3.218)	

Note: * *p* < 0.01, ** *p* < 0.05.

**Table 5:** Linear regression models on school satisfaction (Model 1) and Hayes linear regression considering dispositional gratitude as a moderator variable (Model 2).

Dependent Variable = School Satisfaction (T2)	Model 1		Model 2	
	B	t	B	t
Cyberbullying (T1)	−0.41 *	−2.14	−0.44 **	−2.25
Dispositional Gratitude (T1)	0.16 *	1.98	0.17 *	2.04
Cyberbullying (T1) × Dispositional Gratitude (T1)			0.29	0.83
R2	0.04		0.04	
F (df1,df2)	4.06 (2219)		2.93 (3218)	

Note: ** *p* < 0.01, * *p* < 0.05.
